# Supplementary material for: Likelihood-based approach to discriminate mixtures of network models that vary in time
Source: Sci Rep. 2021 Mar 4;11:5205. doi: 10.1038/s41598-021-84085-0 (PMC7933268; doi:10.1038/s41598-021-84085-0)
Supplement: Supplementary file 1 — Supplementary Information. [file 41598_2021_84085_MOESM1_ESM.pdf]

Supplementary information for Likelihood-based approach to discriminate mixtures of network models that vary in time

Naomi A. Arnold Raul J. Mondragón Richard G. Clegg

January 28, 2021

1 Likelihood Calculation

The main text describes the general form of a model likelihood given observations of an evolving graph $G_1, G_2, \ldots, G_t$:

$$l(M|G = g) = \prod_{i=1}^{t} \mathbb{P}(\Delta_i = \delta_i | G_{i-1} = g_{i-1}, M). \tag{1}$$

Here we provide more details for how each term $\mathbb{P}(\Delta_i = \delta_i|G_{i-1} = g_{i-1}, M)$ is evaluated. In all of the data sets used in this paper, the graphs evolve by adding stars. For example, in the arXiv citation dataset the graph evolves when a new paper arrives and it cites other papers in the dataset. The operation model here is a new node arriving and connecting to m existing nodes. In this case then the m nodes of the star are chosen in turn, all without replacement, to avoid self-loops or multilinks. Now, to calculate the exact probability of this operation we must evaluate the probability of each order occurring. So if we want the likelihood that the chosen set of nodes was $\{2, 3\}$ then we must consider the likelihood of picking 2 then 3 and also 3 then 2.

Consider the observation in figure 1, where we observe a new node (4) joining the network by connecting to nodes (2) and (3), and suppose we wish to compare which of two object models, M_1 and M_2, is a better explanation for this observation. As (4) is a new node, the probability we are interested in is the probability of picking nodes (2) and (3) according to models M_1 or M_2. As there is no order on which of the edges $(4, 2)$ and $(4, 3)$ arrived first, we consider the two different orders. Let us imagine we want compare the BA model with the random model for the pictured addition of one node and two links. In general we have

$$l(M|\text{observation}) = \mathbb{P}(\text{pick node 2, then } 3|M) + \mathbb{P}(\text{pick node 3, then } 2|M).$$

For the BA model we get the following likelihood

$$\begin{aligned} l(M_{\text{BA}}|\text{observation}) &= \underbrace{\frac{k_2}{k_1 + k_2 + k_3} \cdot \frac{k_3}{k_1 + k_3}}_{\text{node 2 then node 3}} + \underbrace{\frac{k_3}{k_1 + k_2 + k_3} \cdot \frac{k_2}{k_1 + k_2}}_{\text{node 3 then node 2}} \\ &= \frac{1}{2} \cdot \frac{1}{2} + \frac{1}{4} \cdot \frac{2}{3} \\ &= \frac{5}{12} \end{aligned}$$

and for the random model we get

$$\begin{aligned} l(M_{\text{rand}}|\text{observation}) &= \frac{1}{3} \cdot \frac{1}{2} + \frac{1}{3} \cdot \frac{1}{2} \\ &= \frac{1}{3}. \end{aligned}$$

In this case, therefore, the BA model is more likely (the likelihood ratio of BA to random being $\frac{5}{4}$. If a node connects to a small number of others then each ordering can be considered explicitly in this way. However the

number of orderings goes up quickly with the number of chosen nodes m (the number of possible orderings of node selections being $m!$) and hence, for $m > 5$ we use a sampling procedure to calculate the average likelihood for a sample of possible orderings and multiply this by the number of orderings.

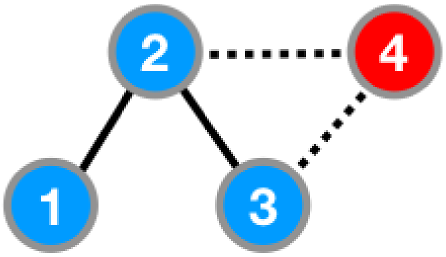

Figure 1: A new node (4) joining the network and connecting to two existing nodes (2,3).

2 Data requirements for the framework

Our framework requires that the history of the network's growth is known to a sufficient temporal resolution such that the edges arriving in each time increment form a star graph. The simplest scenario is if just a single link arrives at each time increment; this is the case for the Facebook wall posts and StackExchange MathOverflow datasets. In many datasets, including all our artificial datasets, each time increment will comprise link arrivals as a star (an existing or new node connecting to more than one other new or existing nodes simultaneously). This is subject to the ordering problem discussed in the previous section, in principle the calculation can be exact but the combinatorics mean that sampling needs to be used. In some data sets more complex situations arise, for example, two unrelated links arriving simultaneously. In principle these could be handled by considering every possible arrival order as well. However, in the data sets we have used these events are extremely rare. They are less than 0.1% of the data and the ordering makes negligible difference to the likelihood calculated so an arbitrary ordering can be assumed without change to the results. Data sets where a large number of unrelated links arrive at exactly the same instant cannot be analysed with this methodology but this still leave a large and increasing number of datasets amenable to analysis.

3 Datasets

This paper used four different publicly available network datasets as case studies for fitting (time varying) mixtures of models. To make these compatible with the modelling framework, we cleaned the datasets to remove any duplicate links and any nodes which do not connect to the largest connected component upon joining the network. These cleaned datasets are available within the software repository [1] in the form of a tab separated file, where each line is of the form

```
SOURCE_NODE    DEST_NODE   TIMESTAMP
```

specifying a string source and destination node for each link and the timestamp (Unix epoch) at which the link was recorded. Table 1 details each dataset showing the original source and number of nodes/edges after cleaning. Also included is a description of what an edge (u, v, t) between nodes u and v created at time t represents for this network. For simplicity we consider these networks as undirected.

4 Extra dataset results

As well as the real data results on figures 8 and 9 with the experiments described in the main text, we generated networks for the remaining Facebook wall posts and citation network datasets. In the case of the citation network (Figure 2), the 'first order' degree based statistics (top row) from the artificial networks

Name	Description and source	Nodes	Edges
Enron emails	Network constructed by a dataset of emails sent or received by Enron employees, where a node is an individual who has emailed or been emailed by an Enron employee. Edge (u, v, t) represents u emailing v at time t. Hosted at `http://konect.cc/networks/enron/` [2]	86978	297456
ArXiV Cit-Hep-Ph	Citation network of papers uploaded to ArXiV under the Physics High-Energy Phenomenology tag between 1993 and 2003. Edge (u, v, t) represents paper u being uploaded at time t citing paper v. Hosted at `https://snap.stanford.edu/data/cit-HepPh.html` [3]	34343	412365
Facebook wall posts	Interaction dataset from wall posts between Facebook users in the New Orleans area over roughly 2.5 years (2006-2009). Edge (u, v, t) represents user u posting on v's wall at time t. `http://konect.uni-koblenz.de/test/networks/facebook-wosn-wall` [4]	45813	183412
StackExchange MathOverflow	Dataset comprising interactions on the MathOverflow forum up to March 2016. An edge (u, v, t) exists if at time t user u answers v's question, replies to v's question or comments on v's answer to a question. `https://snap.stanford.edu/data/sx-mathoverflow.html` [5]	24759	187985

Table 1: Description of datasets used in the paper.

are fairly close to the real data but adding changepoints doesn't seem to add any advantage. The last point is not surprising; there was not much change in the model parameters in the time-varying model (Figure 9 main text). The degree assortativity and clustering coefficient are not realised well by either model. This being said, the model components used for this were deliberately simple to be able to compare the four networks as in Figure 9 main text; if the aim was to find the most realistic model, more complex models such as nonlinear preferential attachment [6] or aging [7] could be considered. For the Facebook wall posts network (Figure 3), the degree-based statistics were well reproduced by both models, with the changepoints not adding much advantage, while the degree assortativity and clustering coefficient were poorly captured by both models. We suggest here that a more realistic model may be achieved by incorporating community structure such as a dynamic variant of the stochastic block model.

5 FETA code for reproducing the experiment in the manuscript

We have provided a Java-based codebase FETA [1] (Framework for Evolving Topology Analysis) for implementing various aspects of this framework, with tutorials provided. The user can generate a network given an operation and object model, calculate the likelihood of a model given network observations (i.e. a dataset of fitting the requirements in section 2), fit a time-varying mixture model to network observations, and extract a time series of different network measurements from a dataset. An API is also provided for users to write their own object models to test on data.

References

[1] N. A. Arnold. FETA: Framework for evolving topology analysis. `https://github.com/narnolddd/FETA3`, 2020.

[2] Bryan Klimt and Yiming Yang. The Enron corpus: A new dataset for email classification research. In *European Conference on Machine Learning*, pages 217–226. Springer, 2004.

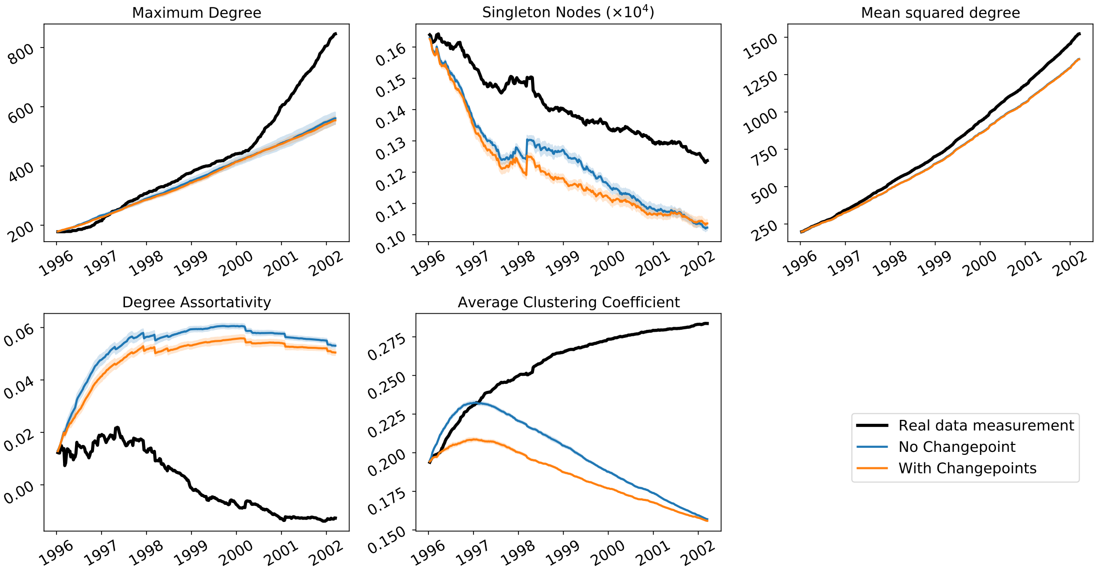

Figure 2: ArXiV Cit-Hep-Ph: Comparison on various network statistics between best fitting mixture model without change and its best fitting counterpart fitted over 10 equally sized intervals for the citation network dataset.

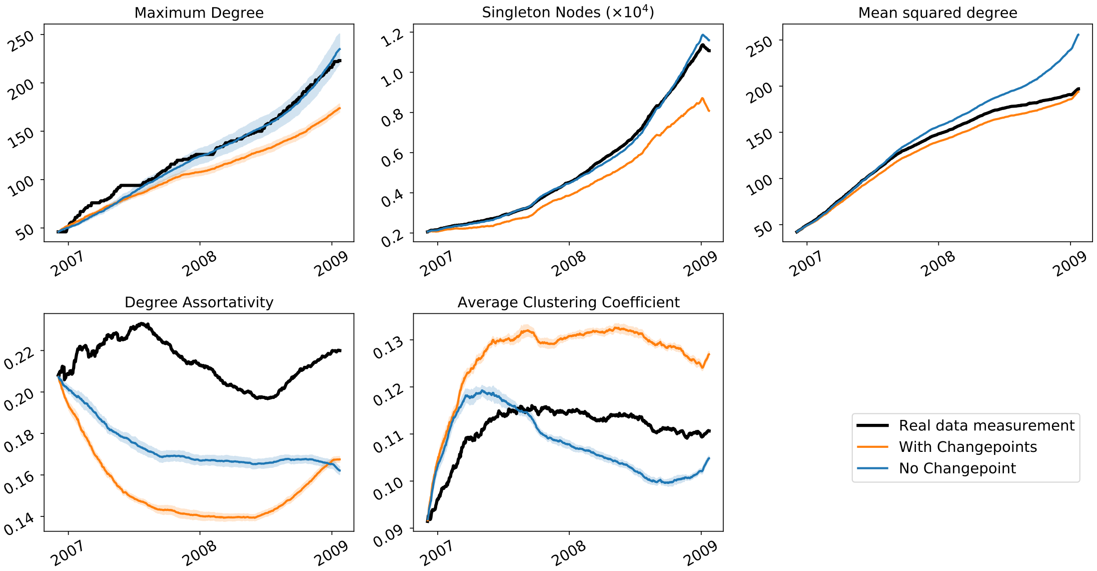

Figure 3: Facebook wall posts: Comparison on various network statistics between best fitting mixture model without change and its best fitting counterpart fitted over 10 equally sized time intervals for the Facebook wall posts dataset.

[3] Johannes Gehrke, Paul Ginsparg, and Jon Kleinberg. Overview of the 2003 KDD Cup. *ACM SIGKDD Explorations Newsletter*, 5(2):149–151, 2003.

[4] Bimal Viswanath, Alan Mislove, Meeyoung Cha, and Krishna P. Gummadi. On the evolution of user interaction in Facebook. In *Proc. ACM Workshop on Online Social Networks*, WOSN '09, pages 37–42, 2009.

[5] Ashwin Paranjape, Austin R. Benson, and Jure Leskovec. Motifs in temporal networks. In *Proc. Tenth ACM International Conference on Web Search and Data Mining*, pages 601–610. ACM, 2017.

[6] Paul L Krapivsky, Sidney Redner, and Francois Leyvraz. Connectivity of growing random networks. *Physical Review Letters*, 85(21):4629, 2000.

[7] Sergey N Dorogovtsev and José Fernando F Mendes. Evolution of networks with aging of sites. *Physical Review E*, 62(2):1842, 2000.
